# Episodic Ultradian Events—Ultradian Rhythms

**DOI:** 10.3390/biology8010015

**Published:** 2019-03-14

**Authors:** Grace H. Goh, Shane K. Maloney, Peter J. Mark, Dominique Blache

**Affiliations:** 1 School of Human Sciences, Faculty of Science, The University of Western Australia, 35 Stirling Highway, Crawley 6009, Western Australia, Australia; grace.goh@uwa.edu.au (G.H.G.); shane.maloney@uwa.edu.au (S.K.M.) peter.mark@uwa.edu.au (P.J.M.); 2 School of Agriculture and Environment and UWA Institute of Agriculture, Faculty of Science, The University of Western Australia, 35 Stirling Highway, Crawley 6009, Western Australia, Australia

**Keywords:** short-term rhythms, temperature, gene, central nervous system, methodology

## Abstract

In the fast lane of chronobiology, ultradian events are short-term rhythms that have been observed since the beginning of modern biology and were quantified about a century ago. They are ubiquitous in all biological systems and found in all organisms, from unicellular organisms to mammals, and from single cells to complex biological functions in multicellular animals. Since these events are aperiodic and last for a few minutes to a few hours, they are better classified as episodic ultradian events (EUEs). Their origin is unclear. However, they could have a molecular basis and could be controlled by hormonal inputs—in vertebrates, they originate from the activity of the central nervous system. EUEs are receiving increasing attention but their aperiodic nature requires specific sampling and analytic tools. While longer scale rhythms are adaptations to predictable changes in the environment, in theory, EUEs could contribute to adaptation by preparing organisms and biological functions for unpredictability.

## 1. Introduction

In Volume 4 of the Handbook of Behavioural Neurobiology, entitled *Biological Rhythms* [1], Jurgen Aschoff defined ultradian rhythms as rhythms with periods shorter than circadian rhythms (that is, less than 24 h). In the same volume, chapters (25 and 26) were dedicated to “Rhythms not directly related to environmental cycles”. The first descriptions of ultradian rhythms were probably made by Symansky in 1920 while studying the behavior of fish and rodents [2]. The early data on ultradian rhythms were mostly behavioral observations, because the technology to continuously monitor physiological parameters was not yet developed. With advances in technology that allows for more sophisticated data collection, ultradian rhythms have now been described for body temperature, blood flow, and many other physiological parameters. In this review, we will first define ultradian rhythms and conclude that they should correctly be called episodic ultradian events. We then review the methods used to sample, detect, and analyze them. Current knowledge of the mechanisms generating episodic ultradian events (EUEs) is discussed including their molecular basis, the role of the central nervous system, neuromediators, and peripheral systems. We also discuss the potential biological role of EUEs. To conclude, we reflect on the future of research on EUEs.

## 2. Ultradian Rhythms: What Are They?

**Figure 1:**
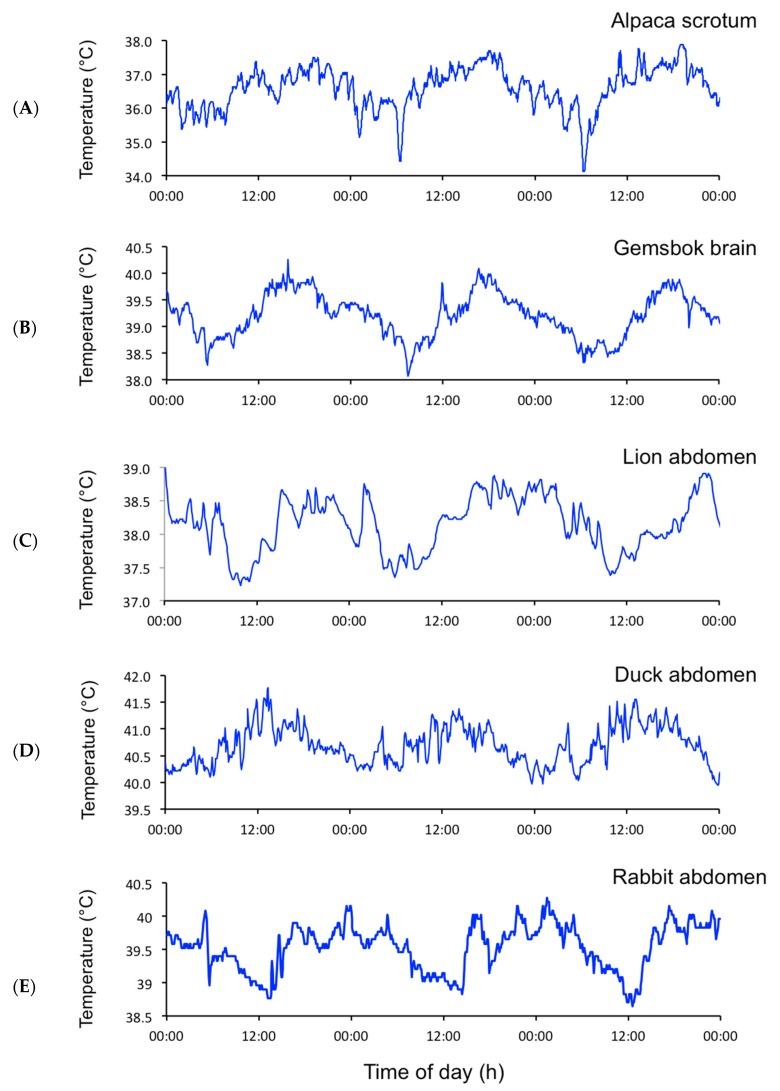
Three-day profiles of temperature in the scrotum of an alpaca ((**A**): sampling rate = 1 min), the brain of a gemsbok ((**B**): sampling rate = 5 min), the peritoneal cavity of a non-pregnant female lion ((**C**): sampling rate = 1 min), a female duck ((**D**): sampling rate = 5 min), and a pregnant female rabbit ((**E**): sampling rate = 5 min). NB: the episodic ultradian events are superimposed on the strong circadian rhythms. All the animals were kept outdoors, either in outdoor paddocks with free access to food and water (**A**,**D**) or in their natural environment [12] (**B**,**C**,**E**). Lion data courtesy of Andrea Fuller [13].

**Figure 2:**
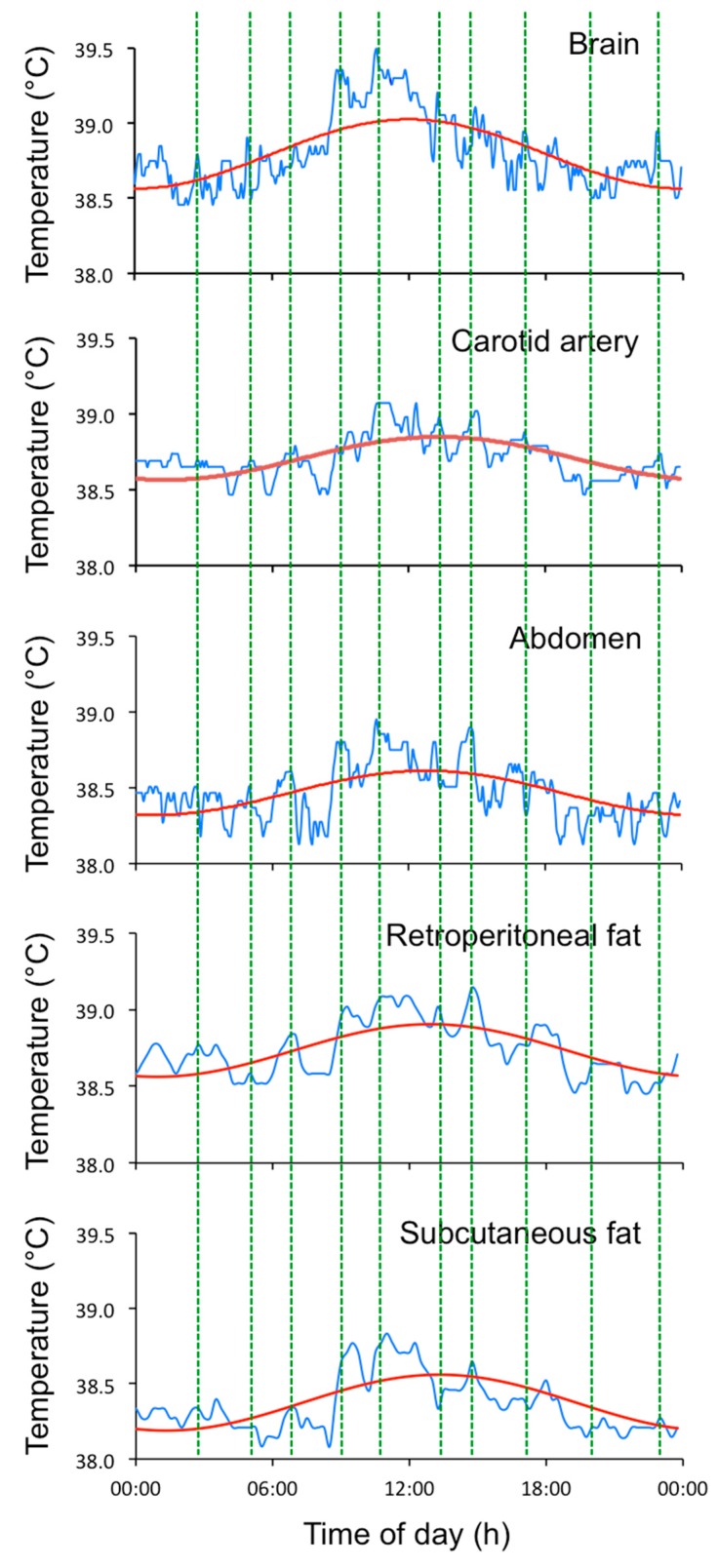
Twenty-four-hour temperature profiles in the brain, carotid artery, abdomen, retroperitoneal fat, and subcutaneous fat of a castrated sheep. The raw data (blue line) were collected from the brain, carotid artery, and abdomen every 1 min and from the two fat tissues every 5 min. The circadian patterns (red line) were fitted to the data using a cosinor analysis [17]. There is a visible synchrony between most of the episodic ultradian events (EUEs) across the different tissues (vertical lines). The sheep was kept indoors under a 12/12 h dark–light cycle and fed at requirement level at 09:00 [12].

Ultradian rhythms (URs) have been defined by Daan and Aschoff as “short-term rhythms with a frequency of 10^−3^ to 5 × 10^−5^ Hz, that is, with periods in the range of 20 min to 6 h” ([3] p 491). Rhythmic activity with shorter periods, such as the patterns of electrical activity of the brain and the heart, will not be discussed. However, in-depth reviews are readily available (for example: [4,5]). Ultradian rhythms in biological processes have been detected in most living organisms and at every level of biological complexity, from eukaryotic microbes [6], e.g., *Caenorhabditis elegans* [7], to metazoans including birds [8] and mammals [9]. Tissues in vivo, and cells in culture, express URs in mass, cell size, protein synthesis, enzyme activity, the concentrations of ATP and numerous hormones, cell respiration and cytoplasmic pH [9]. At the organism level, URs have been reported for body and organ temperatures, carbon dioxide production (V(dot)CO_2_), oxygen consumption (V(dot)O_2_), blood pressure, hormone secretion, urine and feces excretion, digestion, and the phases of sleep [9,10]. Following the publication by Blessing and Ootsuka [11] that dismisses the notion that short-term fluctuations in body temperature are “noise”, we identified ultradian rhythms of temperature in every species of bird and mammal that we measured during our extensive studies on thermoregulation and circadian rhythms (see Figure 1 for examples of the EUEs of core temperature in different species and Figure 2 for an illustration of the EUEs of temperature in different tissues).

As indicated by the title of Part IV of Aschoff’s book, “Rhythms not directly related to environment cycles” [1], URs are often categorized as the outlaws of biological rhythms. Aschoff commented “the great variability in frequency, and the progressive elongation of intervals observed in these rhythms (citing [14]), render their interpretation difficult” ([1], p7). While circadian rhythms are known to be generated by a gene expression-based clock with a period of around 24 h, ultradian rhythms cannot be explained by the interplay of uncoupled circadian clocks of identical or different periods, or short-lived transient ripples [15]. All of these theoretical origins of ultradian rhythms have been discussed in depth, and dismissed, predominantly from work on the behavior of *Drosophila* [15]. For many reasons, the term “ultradian rhythms” is inappropriate for the phenomena because URs are aperiodic (Figure 2). The term “episodic ultradian events” describes the phenomena more accurately [11], as does the term “basic rest-activity cycle” (BRAC) [16]. The terms are similar and EUE will be used here. The fact that behavioral rest and activity cycles occur at time scales similar to many physiological cycles might provide clues to the origin of the timing.

### 2.1. Data Collection and Analysis of EUEs

**Figure 3:**
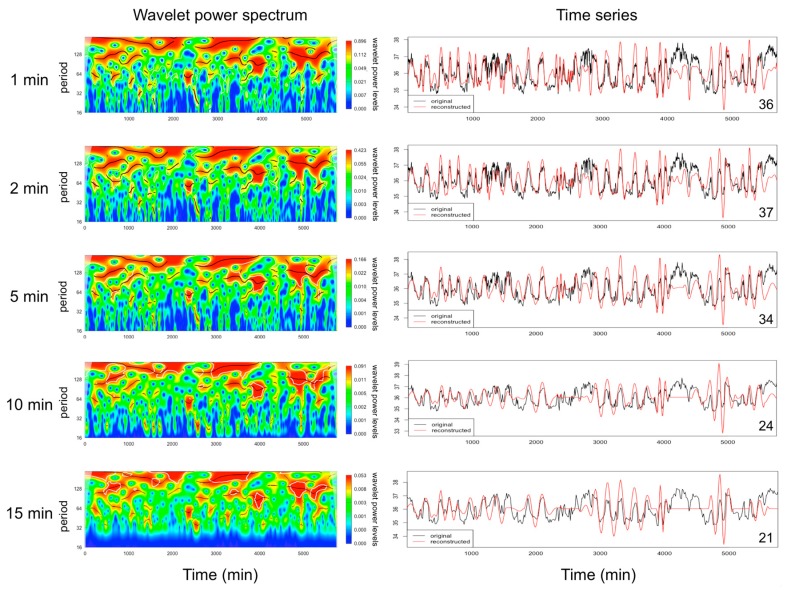
Wavelet power spectrum (left) and time series (right; original data and reconstructed from the wavelet analysis) of a three-day profile of body temperature measured in the abdomen of a mouse sampled at different frequencies based on [24]. Wavelet analysis revealed a loss of EUEs when the sampling interval was longer than 5 min. NB: the number of detected EUEs is indicated in the bottom right corner of the right profiles.

Episodic ultradian events have variable periods ranging from 20 min to a few hours, durations of a few minutes to several hours, and amplitudes that can be quite small compared to the amplitude of other biological rhythms. Therefore, frequent sampling using instrumentation that has good discriminatory power is required to study EUEs. It is impossible to describe a universal methodology to capture EUEs and consideration should be given by researchers according to the nature of the EUEs they study. Blessing and Ootsuka commented that “it is important to examine the record from each individual animal” [11]. Even for a single biological parameter, the frequency of EUEs can vary depending on the environmental or physiological status of the subject. For example, in sheep, the frequency of the episodic secretion of luteinising hormone (LH) can vary 2- to 4-fold depending on the level of nutrition, the level of fatness [18,19], and the time in the oestrus cycle [20]. Similarly, in female sheep, the frequency of LH secretion varies with the hormonal status [21]. To quantify the frequency, duration, and amplitude of episodic events of LH in sheep, blood sampling every 10 to 20 min provides sufficient discrimination [22]. A similar sampling frequency is adequate to detect the episodic nature of other hormones that are secreted/or driven by the pituitary, such as prolactin and cortisol (See [23]). For the EUEs of body temperature in rats during the dark phase, the interval between ultradian events can vary from less than 50 min to longer than 4 h, with the most frequent intervals being 50 to 100 min (see figures 5 and 6 in [11]). A sampling frequency of 1 min should capture all of the information for ultradian events (e.g., locomotor activity, feeding behavior, temperature [11]) that are not direct recordings of cellular electrical signals. The impact of various sampling frequencies on the profile of body temperature is shown in Figure 3, illustrating that the EUEs in the core body temperature of mice are best quantified with a 5 min sampling interval or lower. Wavelet analysis of the EUEs of temperature data is not discriminative when the sampling rate is greater than 5 min (Figure 3).

While EUEs can easily be observed in a time series of biological events, their quantification using statistical analysis is dependent on the characteristics of the signal, such as the shape of the events, the regularity of the intervals between events, and the underlying presence of a circadian rhythm. Episodic events, such as pulses in biological fluids, in serum or plasma, are analyzed using algorithms developed more than 30 years ago, including Pulsar analysis [25], cluster analysis [26], PREDETEC [27], ULTRA [28], and more recent methods based on stochastic models [29]. These methods detect “pulses”, which have a very well-defined shape made up of a rapid rise to a peak within 10 min, followed by an exponential-type return to baseline concentration [30]. Recent advances in computer power have facilitated the use of advanced time series analyses, which has been proposed as a method to detect and quantify EUEs. While periodogram and cosinor analyses are very well suited to the analysis of circadian rhythms [31], the use of these methods at different values of tau, as in Le Fur et al. [32], is often not appropriate because EUEs do not always occur at a regular period [11]. Similarly, Fourier transformation is not recommended because the pattern of individual EUEs is rarely sinusoidal or composed of independent sinusoids, and so cannot be decomposed into a set of frequencies. The most recent mathematical method used to describe EUEs of complex shapes is the wavelet transformation, which analyzes a time series using wavelets, or “mini-waves”, to detect EUEs [24]. Wavelet transformation uses time-limited wavelets of given shapes that fit the EUEs better than a Fourier Transformation that uses infinite waves (sin or cos functions). It has also been proposed that fractal analysis could provide a useful method to explore the complexity of EUE profiles [11]. Recently, dynamic time warping and sparse representation classification have been used to study the pattern of birdsong [33], suggesting that similar mathematical approaches could be used to analyze EUEs. The analysis of frequently sampled data with the statistical tools described above will help to investigate further the origin, the biological role, and the regulation of EUEs, all of which are still very much unknown.

## 3. Origins and Mechanisms Driving EUEs

As already highlighted, EUEs tend to occur at erratic frequencies, which makes their underlying regulatory mechanisms difficult to dissect in the laboratory. Therefore, the mechanisms behind the origin and regulation of EUEs are not as well studied as those of circadian rhythms. Nevertheless, accumulating evidence suggests that the generation of EUEs has a cellular basis.

A recent in vivo study of mice kept in constant darkness showed that in the suprachiasmatic nucleus (SCN), regarded as the master clock for circadian rhythms, there were ultradian fluctuations in the expression of the core clock genes, *Per1*, PER2, and *Bmal1* [34]. Bioluminescent recordings taken every 1 min through an optical fiber at the suprachiasmatic nucleus of *Per1-luc*, PER2::LUC, and *Bmal1-ELuc* transgenic animals revealed ultradian fluctuations with a period of approximately 3 h that were superimposed over the normal circadian rhythm of gene expression. However, the authors reported no stoichiometric correlation between the rhythm of the ultradian clock genes and the rhythms of physical activity of the mice, suggesting that the rhythms of physical activity may be the result of extra-SCN input. At this stage, no follow-up studies have pursued the role and origin of these ultradian clock gene rhythms; further research is critical to elucidate their function and advance research in the field of EUEs. 

Nevertheless, numerous animal models of perturbations in environmental influences or genetic circadian clocks show exaggerations in the frequency of ultradian bouts of activity, arousal, body temperature, and corticosterone secretion [35,36,37,38,39,40,41,42,43]. The fact that these EUEs persist despite the removal of potential *zeitgebers* such as light, food access, and sleep suggests that the EUEs are endogenously generated, rather than behaviorally or environmentally generated [11,42,44,45,46]. These data support the proposition that EUEs are independent of *zeitgebers* and not generated by the same molecular clocks that are responsible for the circadian rhythms. 

### 3.1. Mechanisms of Cellular Ultradian Oscillators

Functional ultradian oscillators certainly exist at the cellular level. Isomura and Kageyama wrote a comprehensive review on the molecular basis and regulation of relatively well-studied ultradian cellular oscillators [47]. They describe four oscillators, namely Hairy and Enhancer of Split transcription factors (*Hes*) *1* and *7* expression, nuclear factor kappa-light-chain-enhancer of activated B cells (NF-κB) signaling activity, p53 activity, and extracellular-signal-regulated kinase (ERK) activity, that all involve negative feedback loops that generate EUEs. In brief, the *Hes* genes in vertebrates encode a family of basic helix-loop-helix transcriptional repressors, the protein product of which inhibits its own mRNA expression [48,49], similar to the way that the CLOCK-BMAL1 protein dimer represses *Bmal1* transcription in the molecular circadian clock and so generates regularly timed oscillations [50]. Unlike the circadian clock machinery, which has several accessory positive and negative feedback loops involving a number of clock genes [51], the *Hes1* and *Hes7* feedback loops appear to consist of only a single negative feedback loop. The short half-life (approximately 20 min) of *Hes* mRNA and its protein product generates a rhythm in *Hes* expression that has a 2-h period [48,52]. In contrast to the circadian clock genes that appear to be expressed in nearly all cells of the vertebrate body, *Hes1* and *Hes7* oscillations appear to be localized primarily to unsegmented, presomitic, mesodermal cells and neuronal stem cells [53] in vertebrates during early embryogenesis, as well as mouse embryonic stem cells [54] and mouse myoblasts [55]. As such, *Hes1* and *Hes7* are unlikely to underlie the coordinated physiological EUEs that are observed in adulthood. However, their rhythms are considered essential in cell fate and vertebrate segmentation respectively [56].

Although the ultradian expression of the *Hes* genes appears relevant only in the developmental context, it is interesting to note the similarities between the mechanisms that drive this ultradian oscillator and the circadian system. The ultradian expression of *Hes1* appears to be cell-autonomous in the cell types where it is endogenously expressed. Interestingly, much like the expression of circadian clocks in peripheral tissues in vitro [57], mouse fibroblast cells transfected with *Hes1* (up to 36 h) and dissociated presomitic mesodermal cells (up to 12 h) show robust, albeit desynchronized, EUEs in vitro [58]. At a population level, *Hes1* expression in dissociated mouse myoblast cells (where *Hes1* is also endogenously expressed) [55] and presomitic mesodermal cells [58] can be transiently rescued through resynchronization by serum shock. Conversely, the ultradian expression of *Hes1* persists in presomitic mesodermal tissue in vivo, and also in dissected (fragmented, but not dissociated) presomitic mesodermal tissue (up to 9 h) in vitro. Taken together, these results suggest that, at a population level, ultradian *Hes1* expression relies on some manner of cell-to-cell communication to sustain synchronized, and therefore physiologically functional, EUEs.

Like the circadian system, the ultradian *Hes1* oscillator is based on a transcriptional–translational feedback mechanism, and the two systems appear similarly regulated. Like many clock genes [59], the period of *Hes1* expression can be transcriptionally regulated through intron splicing mechanisms [56] and microRNA regulation [60]. The amplitude of *Hes1* is also responsive to metabolic regulation through reactive oxygen species, which are by-products of the electron transport chain and NADPH oxidase [61,62]. The treatment of mouse myoblasts with antioxidants led to a significant reduction in *Hes1* expression and a dampening of the amplitude of *Hes1*. Conversely, the amplitude of *Hes1* was increased by the induction of intracellular reactive oxygen species through hydrogen peroxide treatment. Furthermore, this increase in the amplitude of *Hes1* was mediated through calcium signaling [63].

Unlike *Hes* rhythms, the activity of NF-κB, p53, and ERK is not always oscillatory in nature. However, ultradian oscillations in their activity can be triggered by specific factors and accomplished through negative feedback loops involving protein–protein interactions [47]. Tumor necrosis factor alpha (TNFα) stimulates the degradation of inhibitory IκB proteins, which are normally bound to NF-κB complexes. NF-κB then initiates the expression of its target genes, including *IκBα*, which inhibits NF-κB activity, forming a negative feedback loop [64]. A similar negative feedback loop is created for p53 activity upon DNA damage by γ-irradiation. The resulting accumulation of phosphorylated p53 in the nucleus induces the transcription of target genes, including mouse double minute 2 (*Mdm2*), an E3 ubiquitin ligase that contributes to p53 degradation [65]. Oscillatory ERK activity is observed following stimulation by epidermal growth factor [66], TGFα [67], and fibroblast growth factor [68]. Although the exact mechanism that generates ultradian ERK activity is unknown, it is likely that an inhibitory protein–protein negative feedback mechanism is involved [69,70].

### 3.2. A Case for Coupling amongst Ultradian Oscillators

**Figure 4:**
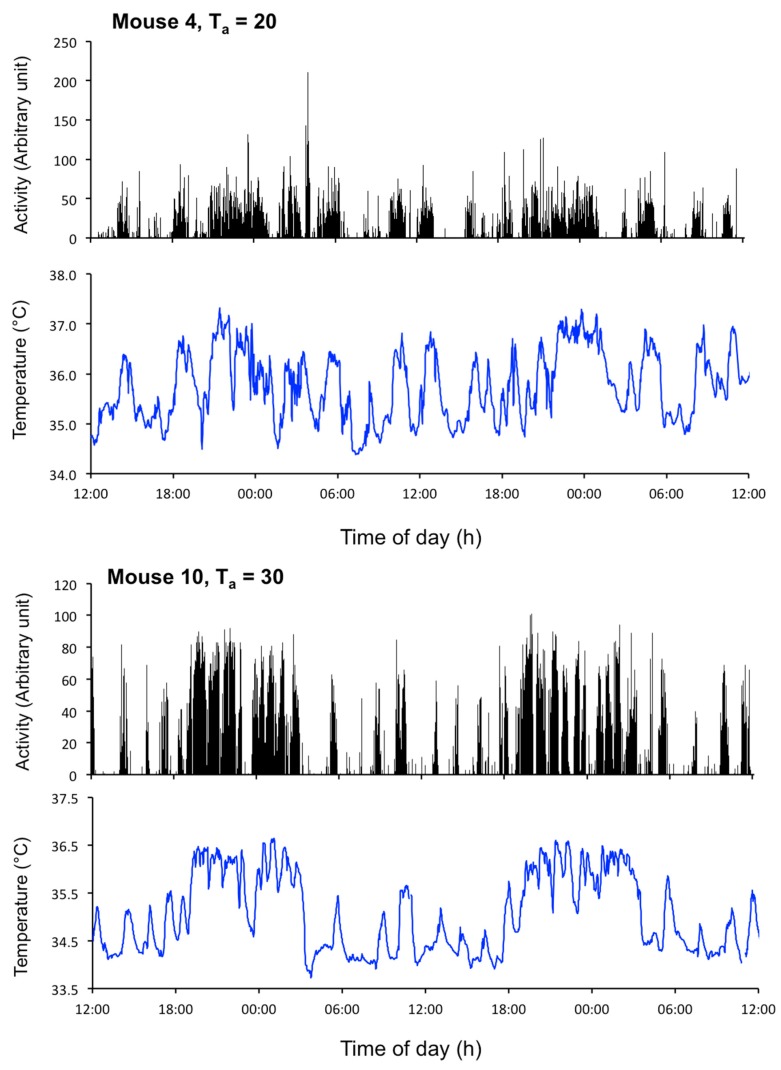
Profiles of activity and body temperature in a mouse kept at an ambient temperature of 20 °C (top two panels) or 30 °C (bottom two panels) to illustrate the synchrony between the EUEs of temperature and the EUEs of activity [79] NB: It has been demonstrated that the EUEs of temperature precede the EUEs of activity [80].

**Figure 5:**
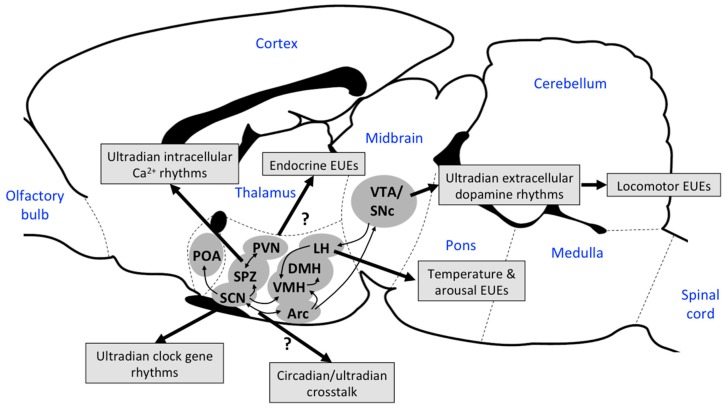
Schematic of the key regions involved in the regulation of EUEs approximated in the rat brain [81]. Major neuronal connections are shown in black arrows and demonstrate a putative network that generates and/or coordinates physiological EUEs. A midbrain dopaminergic oscillator in the VTA/SNc region appears largely responsible for generating EUEs in locomotion [82]. Orexin neurons in the lateral hypothalamus are implicated in generating EUEs of body temperature, arousal and locomotion, but not feeding and heart rate [83]. The pathways connecting the SCN with the ARC and the SPZ may represent sites of crosstalk between the ultradian and circadian systems. The SPZ-PVN region generates EUEs in intracellular calcium levels [84], and EUEs in clock gene expression in the SCN have been observed [34]. The parvocellular and magnocellular neurons in the PVN may regulate endocrine EUEs. However, the functional significance of ultradian clock gene rhythms in the SCN remains unclear.

The mechanisms discussed above that potentially generate cellular EUEs are relevant only in the context of their specific processes. In fact, emerging evidence that several physiological processes such as arousal, body temperature, locomotion, heart rate, feeding behavior, and hormone secretion, are observed to fluctuate in a synchronized manner, at least in rodents and humans [11,42,71,72], makes it apparent that these physiological ultradian events cannot be explained by disparate cell-autonomous feedback mechanisms. For example, Blessing and Ootsuka [11] have convincingly shown that the EUEs in core body temperature are coupled with ultradian peaks in hippocampal electroencephalogram (EEG) power, heart rate, locomotion, and feeding activity in rats (for illustration, see Figure 4). Similarly, the core body temperature is intimately linked to several endocrine axes. In both rodents and humans, the hypothalamic–pituitary–adrenal axis affects thermoregulation by modulating brown adipose thermogenesis [73,74] and, in humans, the EUEs in core body temperature seem to be weakly correlated to salivary cortisol (see Figure 3 in [75]). While there are no studies that link the phase relationship between EUEs in body temperature and the hypothalamic–pituitary–thyroid axis or hypothalamic–pituitary–gonadal axis, hormones in both of these axes have well-documented effects on thermogenesis and body temperature [76,77,78]. Taken together, these data argue towards the existence of a global ultradian oscillator that coordinates EUEs across body systems. Furthermore, the observation that increases in arousal (measured by hippocampal EEG) precede the EUEs in the other physiological systems indicates neural top-down control of ultradian physiology [71]. While there is not yet scientific consensus on the anatomical location of a master ultradian oscillator, accumulating evidence implicates midbrain dopaminergic neurons, orexin neurons, and perhaps the subparaventricular-paraventricular (SPZ-PVN) region in the coordination of physiological EUEs (Figure 5).

### 3.3. Evidence for the Dopaminergic Ultradian Oscillator

Arousal is controlled by the classical ascending arousal system, which comprises the monoamine neurotransmitters including dopamine, histamine, norepinephrine, and serotonin [85]. Of these, several lines of evidence point towards the involvement of a dopaminergic network in the ultradian oscillator. First, perturbation of dopamine levels and dopaminergic neurons appears to alter the period of locomotion and arousal. Dopamine deficiency in tyrosine hydroxylase-deficient mice [86], lesions to brain regions containing dopaminergic neurons such as the arcuate, paraventricular, and retrochiasmatic nucleus in voles [35], or selectively blocking D_2_ dopamine receptors with haloperidol [82], all result in hypoactivity from the suppression of ultradian bursts of locomotion. Conversely, increasing extracellular dopamine through methamphetamine treatment [82] or by genetic ablation of the dopamine transporter, *Slc6a3* [82,87,88,89], results in hyperactivity due to the lengthening of the ultradian period of locomotion. Furthermore, methamphetamine treatment lengthens the ultradian period in a dose-dependent manner [82]. Based on the results of studies using methamphetamine, a suggestion has been made that there is a separate, SCN-independent, circadian oscillator [90]. When taken in the context of recent findings, the putative methamphetamine-associated circadian oscillator could in fact be the ultradian dopamine oscillator with an extremely lengthened period [82]. Second, extracellular dopamine levels in the striatum and midbrain of *Bmal1* knockout mice fluctuate in synchrony with the magnitude and duration of the EUEs of locomotion [82]. Clock-compromised (*Bmal1* knockout) animals were used in those experiments to remove any potential masking effects of circadian rhythms in vivo. Nevertheless, repeating the study in intact animals would confirm whether these extracellular dopamine fluctuations are physiologically relevant for locomotor rhythms in animals with a functional clock, and investigate to what extent, if at all, *Bmal1* rhythms mask EUEs. Third, chemogenetic activation of dopaminergic neurons in the ventral tegmental area (VTA) and the substantia nigra (SN) increases extracellular dopamine levels, leading to a lengthening of ultradian periods, thus phenocopying the effects of dopamine transporter removal and methamphetamine treatment [82]. Lastly, the connectivity of midbrain dopaminergic neurons offers a plausible mechanism for ultradian output and interaction with the circadian system. Neurons from the region approximating the arcuate nucleus (ARC) project to the VTA and the substantia nigra pars compacta (SNc) [91], corroborating findings that lesions in the ARC shorten ultradian periodicity [92]. In addition, orexin neurons in the lateral hypothalamus receive input from the VTA [93] and are responsive to dopaminergic input [94], suggesting that orexin could function as an effector of ultradian output. Studies in mice with chemically ablated orexin neurons and in orexin knockout mice suggest that orexin modulates the amplitude of the EUEs of body temperature (through modulation of brown fat metabolism), locomotion, and arousal [83,95]. However, the EUEs of feeding and heart rate persist in orexin knockouts [83], suggesting that a separate neural pathway activates these systems. 

Although SCN perturbation does not appear to cripple the ultradian oscillator, the ultradian and circadian systems nevertheless interact with each other. In SCN-intact voles kept in constant darkness, the EUEs of feeding and locomotion are phase-locked to the relative active phase of the vole’s circadian rhythm. However, in SCN-lesioned voles in constant darkness, those EUEs are distributed throughout the 24-h period [92]. Likewise, the EUEs in SCN-intact rats tend to occur during their active phase [71,80]. In human newborn infants, the EUEs of activity occur regularly with a 2–6 h period over 24 h. However, activity becomes more concentrated in the active (light) phase as circadian rhythms develop postnatally [96]. 

Dopaminergic neurons in the VTA and SNc do not appear to receive any direct input from the SCN [97], which corroborates multiple findings that SCN lesions do not affect the EUEs of body temperature or locomotion [92,98,99]. The ARC may represent a site of ultradian–circadian crosstalk, because it has reciprocal connections with the SCN [100] and projects to the VTA/SNc [91,101].

In summary, midbrain dopaminergic neurons comprise a key component of the ultradian oscillator for the locomotor period but it is unclear whether those neurons function as a global regulator of physiological EUEs. Critically, the phase relationship of the activity of these neurons with other ultradian parameters, such as body temperature and hormone secretion, has yet to be ascertained. The anatomical connectivity of the putative dopaminergic oscillator only partially explains how some, but not all, EUEs might be generated. Lastly, the cellular and/or network mechanism that is responsible for generating the fluctuating dopaminergic levels remains unknown (Figure 5).

### 3.4. Other Neuronal Mechanisms Driving EUEs

A recent in vitro study of hypothalamic slices containing the SCN [84], SPZ, and PVN of neonatal mice revealed synchronous ultradian fluctuations of the intracellular calcium levels in the PVN, SPZ, and a dorsal part of the SCN. These calcium rhythms had a period of 0.5–4 h and were dependent on glutamate and gamma-aminobutyric acid (GABA) signaling. The SPZ/PVN region appears to be the source of these intracellular calcium EUEs, because slices that contained both the SPZ and the PVN showed only EUEs, whereas slices of isolated SCN showed only circadian rhythms [84]. In addition, tetrodotoxin decreases the amplitude of ultradian calcium rhythms at the population level, but does not affect the calcium rhythms of individual cells [84]. Those results suggest a role for cell-to-cell communication in the maintenance of ultradian synchrony, as has been observed in circadian synchrony [102]. Interestingly, SCN-SPZ-PVN slices that show circadian rhythmicity tend to exhibit frequent rhythms during the peak of circadian calcium levels, but slices without circadian rhythms show sustained rhythms throughout the observation period. This observation mirrors, at a cellular level, the phase-locked relationship of ultradian and circadian locomotor activity and feeding in rodents [92], and of activity in human infants [96]. Unfortunately, Wu et al. did not investigate clock gene expression in their study, so investigating the phase relationship of these calcium rhythms with the ultradian clock gene rhythms observed by Ono et al. [34] would be of special interest as a future study.

Unlike the midbrain dopaminergic neurons, PVN neurons provide direct input to the control of several endocrine systems and the autonomic nervous system [103]. It is, therefore, conceivable that the ultradian calcium rhythms identified by Wu et al. (2018) have a physiological role in generating ultradian endocrine and electrophysiological rhythms. Conversely, lesions of the SPZ appear to have no effect on the EUEs of sleep and body temperature [104]—rather, the SPZ seems to act as a relay for circadian output from the SCN to other neural systems [105]. Studies of PVN-only and SPZ-only slices could elucidate the origin of ultradian calcium rhythms, and provide clues about the mechanism of EUE generation (i.e., whether it is more likely to be a cell ensemble or a cell-autonomous one).

So far, several key regions and pathways in the regulation of EUEs have been identified. However, it is unclear how these rhythms might generate synchronized physiological EUEs. Clearly, further research regarding whether (and how) these systems work together to generate physiological EUEs is warranted (Figure 5).

## 4. The Role of EUEs

Ultradian events in biological activities have often been dismissed as background noise, despite the recognition of their existence and questioning of their significance, by eminent biologists such as Claude Bernard [106], Walter Cannon [107], and even Charles Darwin [108]. Biologists often consider physiological and behavioral changes as responses to environmental stimuli that are the result of a regulatory loop consisting of sensors, effectors, and feedback mechanisms that represent the foundations of homeostasis [107]. As well as such changes that occur acutely in response to environmental triggers, many (perhaps all) biological processes exhibit variation at regular intervals that are independent of acute environmental stimuli, presenting as cyclic activity with different periods [109].

While the homeostatic value of seasonal and circadian rhythms is clear because they adapt animals to changes in the environment, the biological significance of ultradian episodes has been, at best, questioned, and at worst, neglected as irrelevant [11]. At the beginning of this review, we noted that Aschoff described ultradian rhythms in a section about rhythms that are not related to environmental cycles, which means that EUEs could be considered as free-wheeling events, generated randomly and not related to environmental influences. If that were true, the role of EUEs would be difficult to study and their biological utility would be questionable. On the other hand, the previous section described how some genes, some areas of the brain, and some hormonal pathways can modulate the oscillations or the amplitude of EUEs. If a biological mechanism is responsible for the generation of EUEs, it seems highly probable that EUEs have biological value.

What the biological relevance of EUEs actually is remains an open question. There is some consensus in the literature on the adaptive value of EUEs, at least for the EUEs of activity and other behaviors [110]. Like other biological rhythms, the functional significance of EUEs might be in optimizing biological activities [111] by

(1) synchronizing compatible processes, and preventing the simultaneous activation of incompatible processes [10];
(2) preparing biological systems to respond to stimuli such as cell–cell communication [112], and the maintenance of neuronal integrity and alertness [113];
(3) interacting with circadian rhythms.

In the next section, we discuss each of these potential roles for EUEs.

### 4.1. EUEs and the Synchronization of Compatible Processes

Hormonal EUEs are responsible for signaling between cells, organs, and systems. In this role, EUEs can be thought of as the Morse codes of biology. The EUE is an efficient form of coding, since the frequency (and possibly the amplitude) of EUEs can provide information. This encoding is energy efficient, since it does not require a constant signal production and it is less sensitive to noise than threshold-type signaling and to “short blank periods” when the signal is lost for any reason. The reproductive system is an example of the efficiency of EUE coding, a system that achieves the control of ovarian activity and synchronizes physiological and behavioral events, as well as the synchronization of reproductive activity with environmental changes and cycles, all to optimize reproductive success [21,114]. Other hormonal EUEs could be responsible for the synchronization of many different systems. The episodic aspect of (neuro)endocrinology, apart from GnRH, has received relatively little attention, mainly due to the methodological limitations of obtaining frequent samples for long time periods. The low blood volume of small laboratory animals precludes the study of hormone profiles, while the use of large animals, such as sheep or humans, is very time consuming and expensive due to the care of the animals and the number of samples required (288 samples per animal for a 24-h profile with a 5 min sampling frequency). The development of multiplex hormonal assays might open the door to such studies. In fact, recently a coupled oscillator model has been proposed to explain the synchronization between different hormones of the same endocrine system and also the relationship between endocrine axes, such as the hypothalamo–pituitary–adrenal axis and the hypothalamo–pituitary–gonadal axis [72].

It has been proposed that EUEs are responsible for the optimization and restoration of energy supply and use in the body [115,116]. The work of Blessing and Ootsuka and their colleagues has shown that events such as the activation of brown fat and subsequent increase in body temperature precedes, rather than follows, other EUEs such as EUEs of locomotion or feeding, whereas it was previously thought that the activity resulted in a rise in temperature [11,80,117,118]. The EUEs of temperature seem to orchestrate biological function through an energetic link (of temperature) without having a “hard-wired” connection through endocrine or neuronal systems. This role of the EUEs of temperature still needs to be investigated, potentially by using experimental manipulation of EUE frequency. It is possible that a disturbance of EUEs, for example by fever during the early response to pathogens, could be part of the synchronization of the response of other bodily functions.

### 4.2. Role of EUEs in Preparedness

It has been proposed that EUEs are important to the adaptation of organisms [9] and the survival of species because they underlie the preparedness of animals to respond to, for example, fear or predation [10]. EUEs prepare the animal to respond to predator attack [119], and the aperiodicity of behaviors such as feeding, locomotion, or defecation provides a strategy to prevent predation because predators cannot predict the next move of their prey. In fact, a common advice given to people to avoid stalkers is to avoid routine ([120], p69). A role for EUEs in the randomness of activity does not negate the adaptive value of the longer-term routines, such as the circadian rhythms of sleep.

When animals receive a stimulus, such as the detection of a predator, EUEs could contribute to preparing the animal to respond to the stimulus by increasing alertness. A role in alertness seems to hold for the EUEs of body temperature since an increase in temperature by a few tenths of a degree will activate the overall metabolism of every cell that is exposed to the temperature increase. The EUEs are transient events that consist of first an increase in body temperature, then a phase of a decrease in temperature to initial values, followed by a phase of constant temperature lasting until the start of the next EUE, all superimposed on the underlying circadian variation in core body temperature. To test the role of EUEs in the preparedness to respond, it is important to consider the timing of any stimulus. There are a few possible hypotheses regarding when, during an EUE, the brain will be the most responsive to a stimulus. It seems logical that the system would be most responsive during the ascending phase of the EUE or at its peak, because synapses should be at their most active when brain temperature is high [113]. The availability of real-time telemetry recording of body temperature would permit experiments to answer this question, as long as alertness or preparedness can be tested using a rapid test.

### 4.3. EUEs and Circadian Rhythms

In Chapter 15 of *Biological Rhythms*, Daan and Aschoff wrote “These short-term rhythms differ from the circarhythms in that they do not correspond with any known environmental periodicity” ([3], p491). They did not imply that EUEs and circadian rhythms were independent; Aschoff himself suggested some interaction between EUEs and circadian rhythms ([121], p324). During ontogeny, EUEs are present before circadian rhythms are established [121]. In fact, at least in Japanese quail, clearer circadian rhythms in the adults were predicted by a stronger EUE pattern during ontogeny (as defined by [122]). Further, the robust pattern of EUEs was associated with a faster growth rate [122]. More recently, the interaction has been discussed by a few authors [15,72]. A recurring comment is that the frequency of EUEs is inversely related to the strength of the circadian rhythm, as measured by its amplitude [15]. The amplitude of the circadian rhythm seems to be related to the level of challenge that is faced by an animal (For review, see [123]). However, the role of EUEs in modulating the amplitude of the circadian rhythm has not yet been demonstrated.

## 5. Conclusions: What Next in the Field of EUEs?

To understand EUEs, the research effort should be directed towards understanding

(1) the origin of EUEs and further exploration of the molecular basis of EUEs;
(2) the regulation of EUEs by neuromediators such as serotonin and dopamine, and neurohormones such as orexin and kisspeptin;
(3) the relationship between EUEs and other biological rhythms, such as circadian rhythms;
(4) the biological importance or biological function of EUEs, from energy optimization, to alertness, or possibly a combination of both—a theory that has often been proposed but rarely tested experimentally.

Some of these goals would be achieved by re-analyzing existing data sets using newer analytical techniques to detect EUEs, as described in Section 2.1. Such an exercise will help to identify the “the diversity and uniformity of ultradian rhythms” [121] and the interplay between EUEs and other biological rhythms and their potential biological role [121]. Targeted experiments using genetically modified animals for specific genes, neurohormones, or receptors, or the manipulation of the activity of localized areas of the nervous system using deep brain stimulation through techniques such as optogenetics [124], should help to unveil the origin and the regulation of EUEs. The development of a validated in vivo model that allows the manipulation of EUEs by specific factors would help to study the role of EUEs in the adaptive response to a challenge.

## Abbreviations

ARC	arcuate nucleus
PVN	paraventricular nucleus
SCN	suprachiasmatic nucleus
SNc	substantia nigra pars compacta
SPZ	subparaventricular zone
VTA	ventral tegmental area
